# Physical activity and cognitive function in adults born very preterm or with very low birth weight–an individual participant data meta-analysis

**DOI:** 10.1371/journal.pone.0298311

**Published:** 2024-02-13

**Authors:** Kristina Anna Djupvik Aakvik, Silje Dahl Benum, Marjaana Tikanmäki, Petteri Hovi, Katri Räikkönen, Sarah L. Harris, Lianne J. Woodward, Brian A. Darlow, Marit S. Indredavik, Stian Lydersen, Paul Jarle Mork, Eero Kajantie, Kari Anne I. Evensen

**Affiliations:** 1 Department of Clinical and Molecular Medicine, Norwegian University of Science and Technology, Trondheim, Norway; 2 Clinical Medicine Research Unit, Medical Research Center Oulu, Oulu University Hospital and University of Oulu, Oulu, Finland; 3 Public Health Unit, Finnish Institute for Health and Welfare, Helsinki/Oulu, Finland; 4 Children’s Hospital, Helsinki University Hospital and University of Helsinki, Helsinki, Finland; 5 Department of Psychology and Logopedics, University of Helsinki, Helsinki, Finland; 6 Department of Paediatrics, University of Otago Christchurch, Christchurch, New Zealand; 7 School of Health Sciences, University of Canterbury, Christchurch, New Zealand; 8 Department of Mental Health, Norwegian University of Science and Technology, Trondheim, Norway; 9 Department of Public Health and Nursing, Norwegian University of Science and Technology, Trondheim, Norway; 10 Department of Physiotherapy, Oslo Metropolitan University, Oslo, Norway; 11 Unit for Physiotherapy Services, Trondheim Municipality, Trondheim, Norway; 12 Children’s Clinic, St. Olavs Hospital, Trondheim University Hospital, Trondheim, Norway; Center of Pediatrics, GERMANY

## Abstract

**Objective:**

Individuals born very preterm (<32 weeks of gestation) or with very low birthweight (<1500g) have lower cognitive function compared with term-born peers. Furthermore, some studies suggest that they are less physically active as young adults than controls, but the relationship between physical activity and cognitive function remains unclear. We performed an individual participant data meta-analysis to examine whether being born preterm/with very low birth weight is associated with physical activity in adulthood and examined if cognitive function mediates this association.

**Study design:**

Cohorts with data on physical activity and cognitive function in adults born very preterm/very low birth weight and term-born controls were recruited from the Research on European Children and Adults Born Preterm, and the Adults Born Preterm International Collaboration Consortia. A systematic literature search was performed in PubMed and Embase.

**Results:**

Five cohorts with 1644 participants aged 22–28 years (595 very preterm/very low birth weight and 1049 controls) were included. Adults born very preterm/very low birth weight reported 1.11 (95% CI: 0.68 to 1.54) hours less moderate to vigorous physical activity per week than controls, adjusted for cohort, age and sex. The difference between individuals born very preterm/very low birth weight and controls was larger among women than among men. Neither intelligence quotient nor self-reported executive function mediated the association between very preterm/very low birth weight and moderate to vigorous physical activity. Results were essentially the same when we excluded individuals with neurosensory impairments.

**Conclusion:**

Adults born very preterm/very low birth weight, especially women, reported less moderate to vigorous physical activity than their term-born peers. Cognitive function did not mediate this association. Considering the risk of adverse health outcomes among individuals born preterm, physical activity could be a target for intervention.

## Introduction

Every year, more than 15 million births worldwide occur preterm, before 37 weeks of gestation [1, 2], corresponding to approximately 1 in 10 livebirths worldwide [3]. About 1 to 1.5% of newborns are born very preterm (VP<32 weeks of gestation) or with very low birthweight (VLBW<1500g) [2]. Being born VP/VLBW is associated with higher risks of physical, cognitive and developmental challenges during childhood which may persist into adolescence and adulthood [4–8].

Physical activity (PA) has benefits for several physical and mental health outcomes over the life course [9, 10], including all-cause mortality, cardiovascular disease mortality, hypertension, type 2 diabetes, cancer and cognitive health [10]. However, few studies have examined the impacts of being born VP/VLBW on PA. Lowe et al. [9] summarized that children born preterm report lower levels of PA compared with term-born controls, yet longer term studies are limited. Three Finnish studies from two cohorts found that young adults born before 34 weeks [11] and adults born with VLBW [12, 13] reported up to 50% less leisure-time PA than their term-born peers.

It is unclear whether less PA may be related to developmental challenges that have been described in the preterm population. One of the most important neurodevelopmental challenges is cognitive impairment, including both global and specific domains of cognitive function such as executive function [14], which involves the ability to plan, initiate and complete a task [15]. Lower intelligence quotient (IQ) and difficulties in executive function have been consistently described in children [14, 16], adolescents [14] and adults [7, 17] born VP/VLBW. In a population-based cohort study of young men born preterm, physical fitness was associated with cognitive function [18].

We performed an individual participant data (IPD) meta-analysis to examine whether being born VP/VLBW is associated with self-reported PA in adulthood and, if so, whether this association is mediated by cognitive function.

## Methods

### Study design

This study was part of the Research on European Children and Adults Born Preterm (RECAP Preterm) project [19]. In addition, we recruited potential cohorts from the Adults Born Preterm International Collaboration (APIC) Consortium. All data were pseudonymized and transferred to the secure RECAP Preterm node of the Norwegian University of Science and Technology (NTNU) under signed grant agreements or data transfer agreements. All studies had received country-specific ethical reviews, with participants providing written informed consent, including approval of data sharing. All adhered to the Declaration of Helsinki. The study was approved by Regional Committee for Medical and Health Research Ethics in Central Norway (2018/310). The Preferred Reporting Items for Systematic Reviews and Meta-analyses (PRISMA) [20] for analyses of individual participant data was followed.

### Eligibility criteria and search strategy

Eligible for inclusion were prospective cohort studies of individuals born VP/VLBW and term-born controls with data on PA along with assessment of IQ and/or executive function in adulthood.

**Fig 1:**
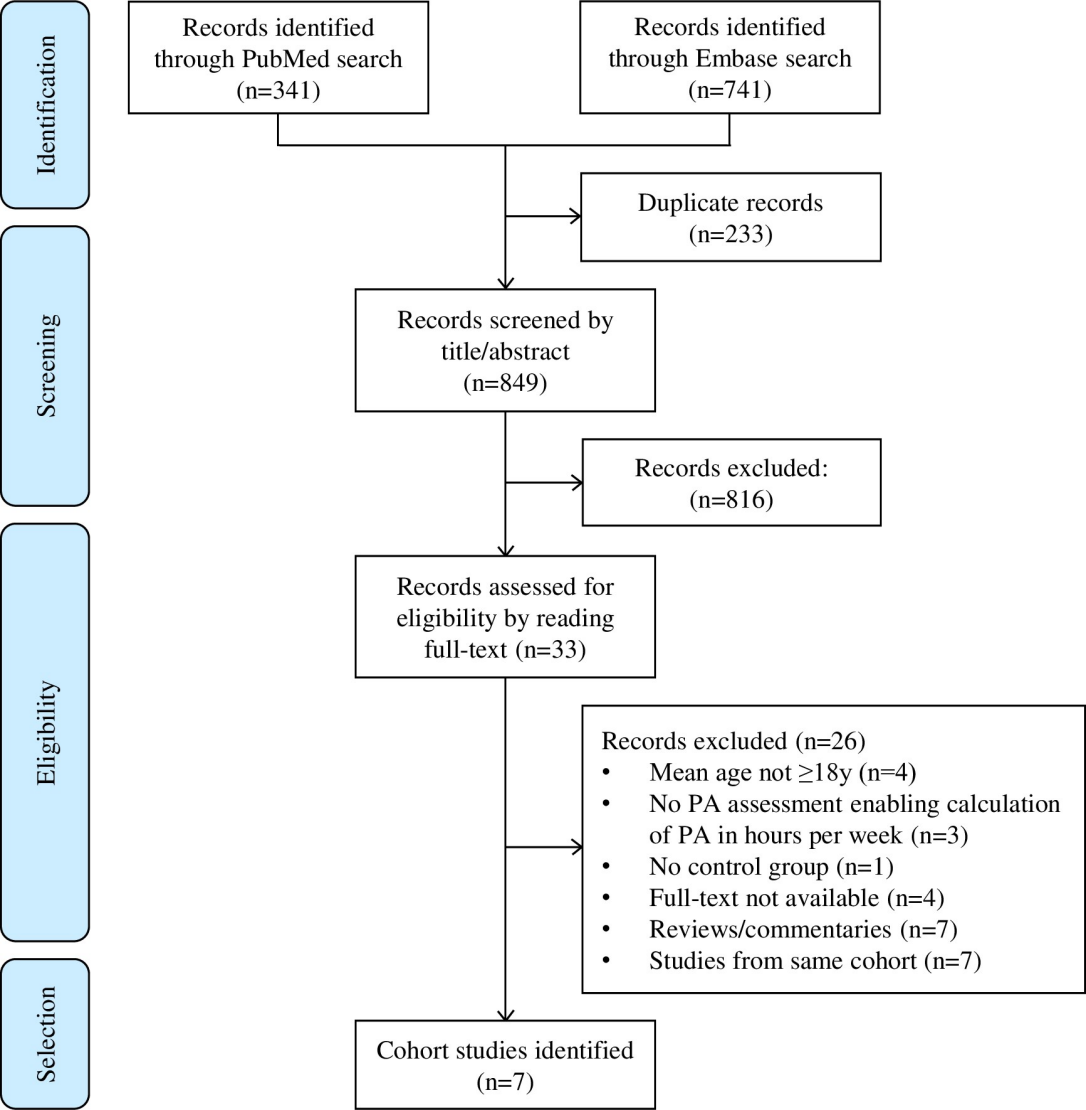
Flow chart for selection of studies from PubMed and Embase. PA = physical activity.

A systematic literature search was performed in PubMed and Embase April-May 2022 by first author (KADA) in collaboration with the NTNU University Library to assess whether there were any additional cohorts with PA assessed in adulthood not already identified through the RECAP Preterm and APIC Consortia. The search was last updated May 4^th^ 2022. Search strategy and flow chart for selection of studies are provided in S1 Table and Fig 1.

### Study selection and background characteristics

Eligibility for inclusion was assessed by two authors (KADA and KAIE). Any disagreements regarding eligibility were resolved by discussion.

Unified criteria were used to define both the VP/VLBW and control group. Background data included individual-level information on demographic and perinatal characteristics, such as birth weight and gestational age, age at follow-up assessment in adulthood, sex and neurosensory impairment (NSI) from childhood. NSI was defined as having one or more of the following: visual impairment, hearing impairment, cerebral palsy or cognitive disability (determined through an IQ test or other information). Bronchopulmonary dysplasia was defined as oxygen supply for ≥28 days or at 36 weeks (postmenstrual age), while intraventricular hemorrhage included grade 1–4. Parental education level was based on highest parental education and was harmonized into low (level 0–2), medium (level 3–5), and high (level 6–8) according to the International Standard Classification of Education.

### Physical activity

Primary outcome was self-reported moderate to vigorous PA (MVPA) assessed by one or several questions related to duration and frequency of PA in each cohort and was harmonized into hours of MVPA per week across the five cohorts (S2 Table). We defined MVPA as activity corresponding to an intensity of ≥3 metabolic equivalents of task (METs) [21]. In the NZ VLBW cohort, one participant was excluded due to extreme values (140 hours of MVPA in a week).

### Cognitive function

Full scale IQ was based on standardized IQ tests (Table 1). Within each cohort, z-scores were calculated [7]. Full scale IQ was restandardized by adding 100 to 15 x z-score, as the mean IQ score is 100 (SD 15) in the general population [7, 22]. A higher IQ score indicates higher cognitive function. The Behavior Rating Inventory of Executive Function–Adult Version (BRIEF-A) was used to assess everyday executive functioning [15]. The BRIEF-A is a standardized self-report questionnaire validated for use in men and women aged 18–90 years. It consists of 75 items providing an overall summary score; the BRIEF-A Global Executive Composite (GEC), which was used in this study. The GEC raw scores were converted into age-standardized scores, with a higher score reflecting poorer executive functioning [15, 17].

**Table 1:** Summary of the cohorts included in individual participant data meta-analysis.

Cohort	Country	Birth year	Initial eligible criteria	Recruitment of term-born controls	Participants^a^/eligible n (%)		IQ test	Age at assessment		
					VP/VLBW	Control		MVPA	IQ	BRIEF-A
AYLS	Finland	1985–86	GA<37 wk (reduced to VP/VLBW for this analysis)	Neonatal period	31/68 (45.6)	332/584 (56.8)	WAIS-III	25y	26y	25y
ESTER	Finland	1985–89	GA<37 wk (reduced to VP/VLBW for this analysis)	Recruited in pregnancy (ESTER NFBC) and adulthood (ESTER non-NFBC)	75/-	345/-	-	23y	-	23y
HeSVA	Finland	1978–85	<1500g	Adulthood	184/254 (72.4)	187/314 (59.6)	WAIS-III	22y	25y	25y
NZ VLBW	New Zealand	1986	<1500g	Adulthood	248/323 (76.8)	100/-	WASI	28y	28y	28y
NTNU LBW Life	Norway	1986–88	<1500g	Infancy	57/82 (69.5)	85/118 (72.0)	WASI	26y	26y	19y

AYLS = Arvo Ylppö Longitudinal Study; BRIEF-A = Behavior Rating Inventory of Executive Function–Adult Version, Global Executive Composite (overall summary score); ESTER = ESTER Preterm Birth Study; GA = gestational age; HeSVA = Helsinki Study of Very Low Birth Weight Adults; IQ = intelligence quotient; MVPA = moderate to vigorous physical activity; NFBC = Northern Finnish Birth Cohort; NTNU LBW Life = Norwegian University of Science and Technology Low Birth Weight in a Lifetime Perspective study; NZ VLBW = New Zealand Very Low Birth Weight Follow-up Study; VP/VLBW = very preterm (<32 weeks of gestation)/very low birth weight (<1500g); WAIS-III = Wechsler Adult Intelligence Scale–Third Edition; WASI = Wechsler Abbreviated Scale of Intelligence; wk = week.

^a^Participants with valid data on moderate to vigorous physical activity.

### Quality assessment

Cohort study quality in terms of selection, comparability and outcome was independently assessed by two authors (KADA and KAIE) using the Newcastle-Ottawa Scale [23]. Assessment criteria were set for each domain, and assessment discrepancies were resolved by discussion (S3 Table).

### Statistical analyses

We analyzed mean differences in MVPA, IQ and GEC scores between the VP/VLBW and control group by using a one-stage approach linear regression with MVPA as dependent variable, group (VP/VLBW vs. control) and cohort as fixed factors, and age and sex as covariates. Interaction with sex was explored by adding the interaction term group x sex. Normality of residuals was judged by visual inspection of QQ-plots. Due to some deviations from normality, we used bootstrapping with B = 2000 bootstrap samples and the bias-corrected and accelerated (BC_a_) method.

**Fig 2:**
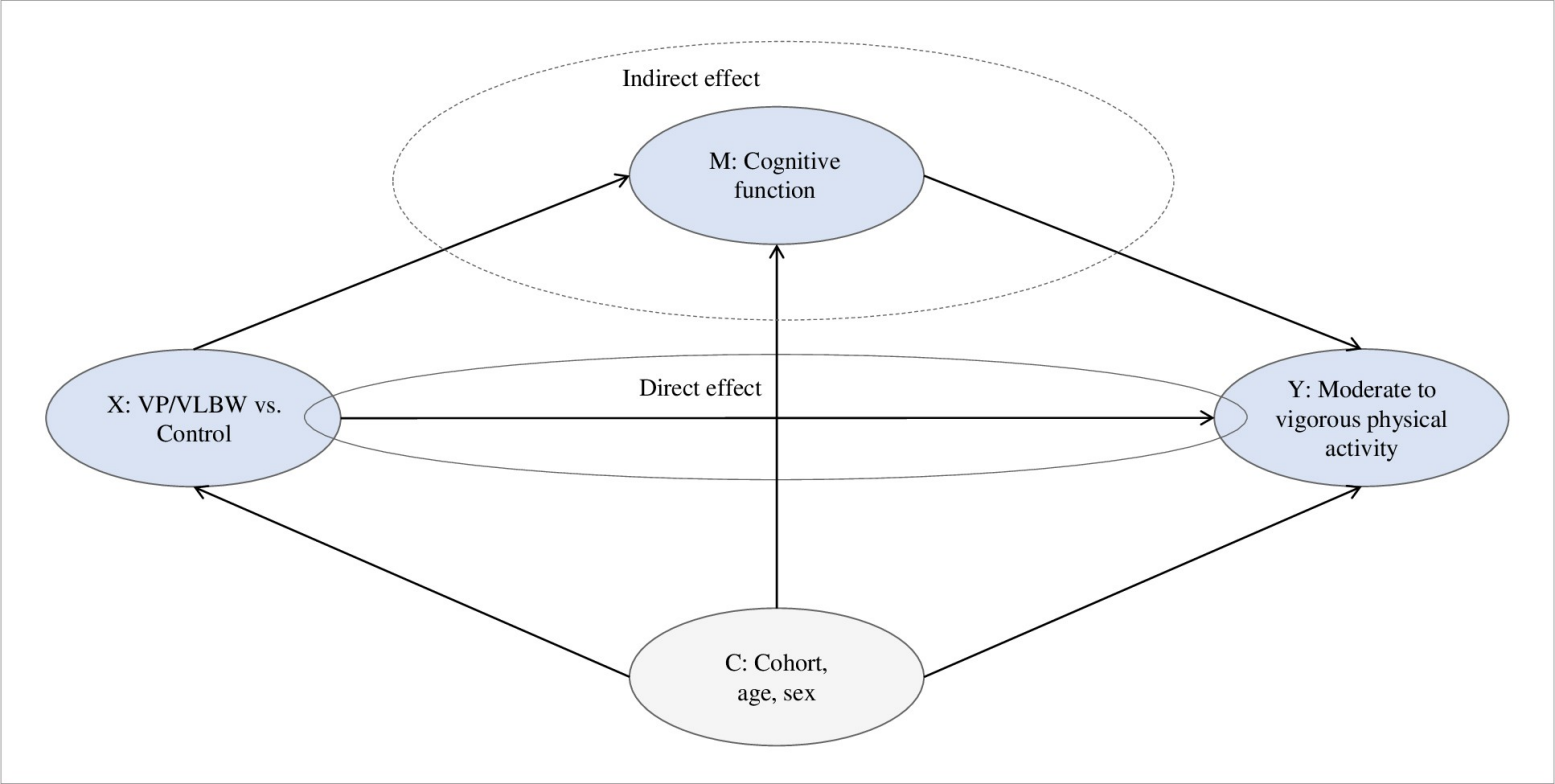
Relationship between group (X), cognitive function (M) and moderate to vigorous physical activity (Y) with confounders (C).

To assess the possible mediating role of cognitive function on the relationship between VP/VLBW and MVPA, we performed mediation analyses using PROCESS macro for IBM SPSS (www.processmacro.org) as developed by Hayes [24] with B = 5000 bootstrap samples. The mediator variables (M), full scale IQ and BRIEF-A GEC scores, were added both separately and combined into the models. Bootstrap confidence intervals were generated for all indirect effects together with possible pairwise comparisons between indirect effects. We entered group as predictor variable (X) and MVPA as outcome variable (Y), and adjusted for cohort, age and sex (C). In Model 1 and 2, IQ and GEC were entered separately as mediators. In Model 3, IQ and GEC were both entered as mediators (Fig 2). As the correlation between these two potential mediators was 0.18, we included both measures simultaneously in the model. We report 95% confidence intervals (CI) where relevant, and we use a significance level of 0.05. Analyses were performed using IBM SPSS Statistics version 27.0.

We performed sensitivity analysis excluding participants with childhood NSI from the total sample and subgroup mediation analyses with participants born extremely preterm (EP<28 weeks of gestation) or with extremely low birth weight (ELBW<1000g) and controls.

## Results

### Study selection and participant characteristics

From the RECAP Preterm Consortium, three Finnish cohorts; Arvo Ylppö Longitudinal Study (AYLS), ESTER Preterm Birth Study (ESTER), and Helsinki Study of Very Low Birth Weight Adults (HeSVA), and one Norwegian cohort; NTNU Low Birth Weight in a Lifetime Perspective study (NTNU LBW Life) were included. From the APIC Consortium, one non-European cohort; New Zealand VLBW Follow-up Study (NZ VLBW) was included (Table 1).

From the literature search, a total of 849 records were screened, 816 records were excluded based on titles and abstracts and 33 full-text articles were assessed for eligibility (Fig 1). Seven cohorts were identified, of which three cohorts were subsequently excluded as we were not able to find records of cognitive assessment in adulthood. The four remaining cohorts were already known through the RECAP Preterm and APIC Consortia. Thus, a total of five cohorts were included in this IPD meta-analysis. All had data on PA measured by self-report and two cohorts also had PA measured by accelerometers [25, 26]. However, as these were in minority, we chose to use self-reported MVPA to obtain a larger sample size.

From the five cohorts, 1644 adults with data on MVPA and cognitive function were included. The VP/VLBW group included 595 participants born <32 weeks of gestation or with birthweight <1500g. The control group included 1049 participants born ≥37 weeks of gestation. Table 2 shows background characteristics of the VP/VLBW and control group in the five cohorts separately and combined. Background characteristics of participants and non-participants in the VP/VLBW and control group are shown in S4 Table.

**Table 2:** Background characteristics of the very preterm/very low birth weight and the control group.

	AYLS				ESTER				HeSVA				NZ VLBW				NTNU LBW Life				Total			
	VP/VLBW		Control		VP/VLBW		Control		VP/VLBW		Control		VP/VLBW		Control		VP/VLBW		Control		VP/VLBW		Control	
	n = 31		n = 332		n = 75		n = 345		n = 184		n = 187		n = 248		n = 100		n = 57		n = 85		n = 595		n = 1049	
Birthweight (g)^a^, mean (SD)	1325	(314)	3618	(470)	1459	(398)	3577	(485)	1130	(215)	3590	(464)	1133	(237)	-	-	1195	(258)	3698	(446)	1189	(285)	3605	(473)
Gestational age (wk)^b^, mean (SD)	29.2	(2.3)	39.7	(1.2)	30.6	(2.1)	40.1	(1.2)	29.2	(2.2)	40.2	(1.1)	29.2	(2.5)	-	-	29.0	(2.6)	39.8	(1.2)	29.4	(2.4)	39.9	(1.2)
Age at assessment (y)^c^, mean (SD)	25.8	(0.6)	25.5	(0.6)	23.0	(1.4)	23.6	(1.1)	22.4	(2.2)	22.4	(2.2)	28.5	(1.1)	28.3	(0.9)	26.3	(0.6)	26.5	(0.5)	25.6	(3.1)	24.6	(2.1)
Female sex^d^, n (%)	14	(45.2)	186	(56.0)	42	(56.0)	181	(52.6)	103	(56.0)	111	(59.4)	142	(57.3)	63	(63.0)	27	(47.4)	50	(58.8)	328	(55.1)	591	(56.4)
NSI^b^^,^^e^, n (%)	6	(19.4)	5	(1.5)	7	(9.3)	*	*	21	(11.4)	0	(0)	20	(8.1)	-	-	7	(12.3)	0	(0)	61	(10.3)	8	(0.8)

AYLS = Arvo Ylppö Longitudinal Study; ESTER = ESTER Preterm Birth Study; HeSVA = Helsinki Study of Very Low Birth Weight Adults; NSI = neurosensory impairment from childhood defined as having one or more of the following: visual impairment, hearing impairment, cerebral palsy or cognitive disability (determined through an IQ test or other information); NTNU LBW Life = Norwegian University of Science and Technology Low Birth Weight in a Lifetime Perspective study; NZ VLBW = New Zealand Very Low Birth Weight Follow-up Study; SD = standard deviation; VP/VLBW = very preterm (<32 weeks of gestation)/very low birth weight (<1500g); wk = week.

^a^Data missing for one control participant in the ESTER cohort.

^b^Data missing for all control participants in the NZ VLBW cohort, but they were born at term (≥37 weeks of gestation).

^c^Data missing for 14 control participants in the AYLS cohort.

^d^Data missing for one control participant in the ESTER cohort.

^e^Data missing for two VP/VLBW and 53 control participants in the AYLS cohort, 23 VP/VLBW and 19 control participants in the HeSVA cohort, eight VP/VLBW participants in the NZ VLBW cohort, 19 VP/VLBW and 26 control participants in the NTNU LBW Life cohort.

*Exact number not presented for counts n<5 to protect the privacy of the participants.

### Quality of included cohort studies

Based on the Newcastle-Ottawa Scale [23], quality scores ranged from 6 to 8 for the included cohort studies (S3 Table), with a mean quality score of 7.0 (SD 0.71). Studies were rated highly on representativeness, ascertainment of exposure and comparability, however all outcomes were self-reported.

### Results of synthesis

Table 3 shows mean self-reported MVPA, full scale IQ and BRIEF-A GEC scores in the two groups. Mean MVPA per week was 3.65 (SD 5.02) in the VP/VLBW group compared with 6.09 (SD 3.27) hours in the control group. Mean difference in MVPA per week adjusted for cohort, age and sex was -1.11 (95% CI: -1.54 to -0.68) hours. There was a significant group x sex interaction (p<0.001). We therefore also performed separate analyses for women and men. Adjusted mean differences in MVPA per week were -1.34 (95% CI: -1.84 to -0.84) for women and -0.86 (95% CI: -1.57 to -0.14) for men (S5 Table).

**Table 3:** Moderate to vigorous physical activity, full scale intelligence quotient and Behavior Rating Inventory of Executive Function–Adult Version, Global Executive Composite in the very preterm/very low birth weight and the control group.

	n	VP/VLBW			Control			n^a^	Adjusted mean difference (95% CI)^b^		p-value
		n	Mean	(SD)	n	Mean	(SD)				
MVPA (hours per week)	1644	595	3.65	(5.02)	1049	6.09	(3.27)	595, 1034	-1.11	(-1.54 to -0.68)	< .001
Full scale IQ	983	407	85.9	(20.0)	576	100.2	(14.7)	407, 564	-14.4	(-17.1 to -11.5)	< .001
BRIEF-A GEC	1356	474	101.9	(22.5)	882	100.5	(20.1)	474, 881	0.2	(-2.4 to 2.9)	0.913

BRIEF-A GEC = Behavior Rating Inventory of Executive Function–Adult Version, Global Executive Composite (overall summary score); CI = confidence interval; IQ = intelligence quotient; MVPA = moderate to vigorous physical activity; SD = standard deviation; VP/VLBW = very preterm (<32 weeks of gestation)/very low birth weight (<1500g).

^a^VP/VLBW, Control.

^b^Based on bootstrapped regression analysis with group and cohort as fixed factor, and age and sex as covariates.

Mean IQ for the VP/VLBW group was 14.4 (95% CI: 11.5 to 17.1) points lower than the control group. The difference in GEC scores between the groups was 0.2 (95% CI: -2.4 to 2.9) (Table 3).

Table 4 shows the results of the mediation analysis with the direct, indirect and total effect in three models. The total effect of the association between VP/VLBW and MVPA, with IQ or GEC score as mediators was -0.79 (95% CI: -1.41 to -0.17 and -1.28 to -0.30) hours per week in single models (Model 1 and 2) and -0.73 (95% CI: -1.38 to -0.07) hours per week in the multiple mediator model (Model 3). The indirect effects of IQ and GEC scores were small and not significant in all three models. Thus, the direct effect of VP/VLBW on MVPA was negative and significant. In separate analyses by sex, the total effect in Model 3 was -1.21 (95% CI: -1.87 to -0.56) hours per week for women and -0.09 (95% CI: -1.33 to 1.16) hours per week for men. The indirect effect of IQ and/or GEC scores in Model 1–3 was not significant for either women or men.

**Table 4:** Direct, indirect and total effect of very preterm/very low birth weight on moderate to vigorous physical activity with cognitive function as mediator.

Model	Cognitive function	n^a^	Direct effect of VP/VLBW		Indirect effect of VP/VLBW		Total effect of VP/VLBW	
			Estimate	95% CI	Estimate	95% CI	Estimate	95% CI
1	Full scale IQ	407, 564	-0.70	(-1.36 to -0.03)	-0.09	(-0.34 to 0.15)	-0.79	(-1.41 to -0.17)
2	BRIEF-A GEC	474, 881	-0.79	(-1.27 to -0.30)	-0.003	(-0.05 to 0.05)	-0.79	(-1.28 to -0.30)
3	Full scale IQ and BRIEF-A GEC	382, 525	-0.72	(-1.42 to -0.02)	-0.008	(-0.27 to 0.25)	-0.73	(-1.38 to -0.07)

Analyses adjusted for cohort, age and sex.

BRIEF-A GEC = Behavior Rating Inventory of Executive Function–Adult Version, Global Executive Composite (overall summary score); CI = confidence interval; IQ = intelligence quotient; MVPA = moderate to vigorous physical activity; VP/VLBW = very preterm (<32 weeks of gestation)/very low birth weight (<1500g).

^a^VP/VLBW, Control.

### Sensitivity and subgroup analyses

Sensitivity analysis excluding participants with childhood NSI showed essentially the same mean differences as for the total sample (S6 Table). The total effect of the association between VP/VLBW and MVPA was negative in all models ranging from -0.59 (95% CI: -1.27 to 0.08) to -0.70 (95% CI: -1.34 to -0.06) hours per week compared with controls (S7 Table).

Subgroup analysis showed that participants born EP/ELBW reported 1.60 (95% CI: 0.89 to 2.25) hours less MVPA per week and had 18.7 (95% CI: 14.8 to 22.4) points lower full scale IQ than controls (S8 Table). The total effect of the association between EP/ELBW and MVPA was negative and the indirect effects of IQ and/or GEC were small and non-significant in all models (S9 Table).

## Discussion

In this IPD meta-analysis we found that adults born VP/VLBW reported about one hour less MVPA than adults born at term. The associations with MVPA were stronger among women and participants born EP/ELBW compared with controls. However, cognitive function did not explain the difference in strength between these associations. Results were essentially the same in sensitivity analyses when we excluded participants with NSI.

This is the first IPD meta-analysis to examine the possible mediating role of cognitive function on MVPA among adults born preterm. Strengths of this study were inclusion of five high quality cohorts supplemented with a systematic literature search, allowing us to perform an IPD meta-analysis with a large sample. The groups were defined by uniform criteria across the cohorts. All cohorts were recruited around the same years of birth and assessed at approximately similar age. Nevertheless, we adjusted for cohort, age and sex in our main analysis, as potential confounders that could impact the results. Even though slightly different questions in the five cohorts may have led to variations in type of PA reported, all cohorts had data available to create a harmonized variable of self-reported MVPA in hours per week. Cognitive function was assessed using reliable and valid, standardized measures, including the Wechsler scales WAIS-III and WASI, and the BRIEF-A self-report rating scale.

Limitations include possible bias due to dropout, as loss to follow-up is inevitable in long-term cohort studies and may lead to overrepresentation of healthier participants [27]. However, most cohorts had less than 50% dropout. All participants needed valid data on the outcome variable to be included in the main analysis and data on at least one of the mediators to be included in the mediation analyses. The ESTER cohort did not have IQ data and was not included in two of the mediation analyses. Another possible limitation is self-report bias due to social desirability [28]. However, this would only be expected to introduce bias if the effects of social desirability were different in the two groups.

There are few studies on self-reported PA in adults born preterm. Three existing studies include the Finnish cohorts, ESTER [11] and HeSVA [12, 13] which found that young adults born early preterm at less than 34 weeks and adults born VLBW reported less PA compared with term-born controls. As these cohorts were included in the present study, this may explain the similar findings, although Tikanmäki et al. [11] and Kaseva et al. [13] reported on PA assessed over a 12-month period including 30 types of PA. Few studies have examined sex differences in PA, however the HeSVA cohort reported no interaction effect of sex. The finding of a larger group difference among women in our increased sample may be in line with other prematurity-associated relative risks disproportionately affecting women, such as coronary heart disease [29], type 2 diabetes [30], hypertension [31] and depression [32].

Adjusted IQ score was 14.4 points lower in the VP/VLBW compared with the control group. This fits well with the 12 points difference in IQ reported in another IPD meta-analysis from RECAP Preterm with a larger number of participants (1068 VP/VLBW and 1067 controls) [7]. Other studies of VP adults have also reported lower scores for intellectual ability and IQ estimates compared with controls [33–35]. We did not find a difference in BRIEF-A GEC score between the VP/VLBW and control group, in line with the results published from the NTNU LBW Life cohort [36]. However, the NZ VLBW cohort reported poorer BRIEF-A scores [17]. In the HeSVA cohort, VP/VLBW adults reported lower GEC scores, indicating better executive function, however parent-reports indicated more problems [37]. Studies using executive function tests, including the HeSVA and NTNU LBW Life cohorts, have reported poorer executive function among preterm born adults compared to term-born peers [33–35, 38, 39].

To our knowledge, no studies have examined the possible mediating role of cognitive function on PA in individuals born preterm. We found that neither full scale IQ nor BRIEF-A GEC scores affected the relationship between VP/VLBW and MVPA in single and multiple mediation models. In a population-based registry study of young men born preterm who were conscripted into military service, physical fitness measured by maximum performance in ergometer cycling was positively associated with higher cognitive function. However, PA was not measured, and cognitive function was assessed by an unstandardized test developed by the Swedish military [18]. In the general population, a recent cross-sectional study with data from more than 6,800 mid-age adults found that physical fitness was associated with better cognitive function, while self-reported PA was not [40].

### Biological plausibility

As we found that cognitive function did not mediate the association between VP/VLBW and self-reported MVPA, explanations for lower MVPA may be related to other factors. One explanation could be deficits in coordination and muscle strength of individuals born very preterm [9]. For example, two review articles have shown that adolescents and adults born VP/VLBW have more motor impairments compared with term-born controls [6, 41]. Secondly and relatedly, motor difficulties could also be associated with visual problems found in adolescence [42] and adulthood [43, 44]. These conditions may affect the ability to perform everyday life functional activities including a physically active lifestyle [9]. Thirdly, Tikanmäki et al. [11] have also suggested reduced muscle mass, lower muscular fitness, lower pulmonary function and poorer self-efficacy as potential explanations. Further studies could explore whether these factors can explain lower self-reported MVPA in VP/VLBW individuals.

### Clinical implications

Even though the World Health Organization 2020 guidelines for PA and sedentary behavior provide evidence-based recommendations for PA across all ages and abilities, the exact dose-response association between volume and/or intensity of PA and health outcomes remains uncertain [10]. However, according to an umbrella review, it does not appear to be a lower dose threshold for PA to induce health benefits, and even small increments in PA can lead to marked health benefits [45].

Our finding of less self-reported MVPA in adults born VP/VLBW could be related to adverse health outcomes in this group. Individuals born VP/VLBW have higher rates of cardiometabolic risk factors [4, 31, 46], pulmonary risk factors [9, 47–49] and manifest diseases like ischemic heart disease [50] and type 2 diabetes [51]. Thus, any intervention which may reduce the risk of these conditions may be substantial [4]. A recent meta-analysis and systematic review on interventions and longitudinal studies, including more than 75,000 healthy adults, found that PA reduces the risk of cardiovascular diseases [52].

As the VP/VLBW group reported that they were less physically active than controls, PA interventions may be relevant for reducing the risk of chronic diseases also in adults born preterm. Increasing PA with even small amounts per weeks could be beneficial, especially of higher intensities as MVPA. This might be even more important for women and individuals born EP/ELBW as they were even less physically active compared with controls. Early encouragement to be physically active and participate in leisure-time PA from young age might be beneficial [53]. Evaluating personal, familial, and environmental factors related to being physically active may help identify preferences and potential barriers [53]. Such knowledge would be helpful in order to understand how individuals born preterm can be motivated to engage in more MVPA.

In conclusion, we found that being born VP/VLBW was associated with less self-reported MVPA in adulthood and this association was stronger among women. However, cognitive function did not mediate the association. Considering the risk of adverse health outcomes among individuals born preterm, PA could be a target for intervention.

## Supporting information

S1 Checklist PRISMA-IPD checklist of items to include when reporting a systematic review and meta-analysis of individual participant data (IPD). (DOC)

S1 Table Search strategy in PubMed and Embase. Search last updated May 4^th^ 2022. / = Emthree term; ti,ab,kw = title, abstract and keywords fields.
(DOCX)

S2 Table Overview of questions used to assess moderate to vigorous physical activity. AYLS = Arvo Ylppö Longitudinal Study; ESTER = ESTER Preterm Birth Study; HeSVA = Helsinki Study of Very Low Birth Weight Adults; NTNU LBW Life = Norwegian University of Science and Technology Low Birth Weight in a Lifetime Perspective study; NZ VLBW = New Zealand Very Low Birth Weight Follow-up Study; Q = question.
(DOCX)

S3 Table Newcastle-Ottawa criteria and quality score for each cohort. AYLS = Arvo Ylppö Longitudinal Study; ESTER = ESTER Preterm Birth Study; HeSVA = Helsinki Study of Very Low Birth Weight Adults; NTNU LBW Life = Norwegian University of Science and Technology Low Birth Weight in a Lifetime Perspective study; NZ VLBW = New Zealand Very Low Birth Weight Follow-up Study.
(DOCX)

S4 Table Background characteristics of participants and non-participants in the very preterm/very low birth weight and the control group. ISCED = International Standard Classification of Education; NSI = neurosensory impairment from childhood defined as having one or more of the following: visual impairment, hearing impairment, cerebral palsy or cognitive disability (determined through an IQ test or other information); SD = standard deviation; VP/VLBW = very preterm (<32 weeks of gestation)/very low birth weight (<1500g); wk = week. ^a^ESTER Preterm Birth Study cohort not included due to no data available for non-participants. ^b^No data available for the control group in the NZ VLBW cohort. ^c^Data missing for 77 participants and 30 non-participants in the VP/VLBW group. ^d^Data missing for 39 participants and 42 non-participants in the VP/VLBW group. ^e^Data missing for 52 participants and 114 non-participants in the VP/VLBW group, and 98 participants and 223 non-participants in the control group. ^f^Data missing for 18 participants and 95 non-participants in the VP/VLBW group, and 17 participants and 141 non-participants in the control group.
(DOCX)

S5 Table Moderate to vigorous physical activity among women and men in the very preterm/very low birth weight and the control group. CI = confidence interval; MVPA = moderate to vigorous physical activity; SD = standard deviation; VP/VLBW = very preterm (<32 weeks of gestation)/very low birth weight (<1500g). ^a^VP/VLBW, Control. ^b^Based on bootstrapped regression analysis with group and cohort as fixed factor, and age and sex as covariates.
(DOCX)

S6 Table Moderate to vigorous physical activity, full scale intelligence quotient and Behavior Rating Inventory of Executive Function–Adult Version, Global Executive Composite in the very preterm/very low birth weight and the control group. Participants with neurosensory impairment excluded. BRIEF-A GEC = Behavior Rating Inventory of Executive Function–Adult Version, Global Executive Composite (overall summary score); CI = confidence interval; IQ = intelligence quotient; MVPA = moderate to vigorous physical activity; SD = standard deviation; VP/VLBW = very preterm (<32 weeks of gestation)/very low birth weight (<1500g). ^a^VP/VLBW, Control. ^b^Based on bootstrapped regression analysis with group and cohort as fixed factor, and age and sex as covariates.
(DOCX)

S7 Table Direct, indirect and total effect of very preterm/very low birth weight on moderate to vigorous physical activity with cognitive function as mediator. Participants with neurosensory impairment excluded. Analyses adjusted for cohort, age and sex. BRIEF-A GEC = Behavior Rating Inventory of Executive Function–Adult Version, Global Executive Composite (overall summary score); CI = confidence interval; IQ = intelligence quotient; VP/VLBW = very preterm (<32 weeks of gestation)/very low birth weight (<1500g). ^a^VP/VLBW, Control.
(DOCX)

S8 Table Moderate to vigorous physical activity, full scale intelligence quotient and Behavior Rating Inventory of Executive Function–Adult Version, Global Executive Composite in the extremely preterm/extremely low birth weight and the control group. BRIEF-A GEC = Behavior Rating Inventory of Executive Function–Adult Version, Global Executive Composite (overall summary score); CI = confidence interval; EP/ELBW = extremely preterm (<28 weeks of gestation)/extremely low birth weight (<1000g); IQ = intelligence quotient; MVPA = moderate to vigorous physical activity; SD = standard deviation. ^a^EP/ELBW, Control. ^b^Based on bootstrapped regression analysis with group and cohort as fixed factor, and age and sex as covariates.
(DOCX)

S9 Table Direct, indirect and total effect of extremely preterm/extremely low birth weight on moderate to vigorous physical activity with cognitive function as mediator. Analyses adjusted for cohort, age and sex. BRIEF-A GEC = Behavior Rating Inventory of Executive Function–Adult Version, Global Executive Composite (overall summary score); CI = confidence interval; EP/ELBW = extremely preterm (<28 weeks of gestation)/extremely low birth weight (<1000g); IQ = intelligence quotient; SD = standard deviation. ^a^EP/ELBW, Control.
(DOCX)
