# Supplementary material for: Physical activity and cognitive function in adults born very preterm or with very low birth weight–an individual participant data meta-analysis
Source: PLoS One. 2024 Feb 13;19(2):e0298311. doi: 10.1371/journal.pone.0298311 (PMC10863878; doi:10.1371/journal.pone.0298311)
Supplement: S1 Table — Search last updated May 4th 2022. / = Emthree term; ti,ab,kw = title, abstract and keywords fields. (DOCX) [file pone.0298311.s002.docx]

**S1 Table. Search strategy in PubMed and Embase.**

| PubMed | | |
| --- | --- | --- |
| Row | Query | Results |
| 1 | "infant, very low birth weight"[Mesh Terms] | 11 314 |
| 2 | "very low birth weight*"[All Fields] | 13 135 |
| 3 | "very low birthweight*"[All Fields] | 2 162 |
| 4 | "premature birth"[Mesh Terms] | 18 209 |
| 5 | "premature birth*"[All Fields] | 22 060 |
| 6 | "preterm*"[All Fields] | 89 646 |
| 7 | "infant, very low birth weight"[Mesh Terms] OR "very low birth weight*"[All Fields] OR "very low birthweight*"[All Fields] OR "premature birth"[Mesh Terms] OR "premature birth*"[All Fields] OR "preterm*"[All Fields] | 105 211 |
| 8 | "exercise"[Mesh Terms] | 230 701 |
| 9 | "exercise*"[All Fields] | 469 595 |
| 10 | "physical activ*"[All Fields] | 147 867 |
| 11 | "exercise"[Mesh Terms] OR "exercise*"[All Fields] OR "physical activ*"[All Fields] | 592 840 |
| 12 | "adult"[Mesh Terms] | 7 788 952 |
| 13 | "adult*"[All Fields] | 6 341 638 |
| 14 | "adult"[Mesh Terms] OR "adult*"[All Fields] | 8 502 363 |
| 15 | "infant, very low birth weight"[Mesh Terms] OR "very low birth weight*"[All Fields] OR "very low birthweight*"[All Fields] OR "premature birth"[Mesh Terms] OR "premature birth*"[All Fields] OR "preterm*"[All Fields] AND "exercise"[Mesh Terms] OR "exercise*"[All Fields] OR "physical activ*"[All Fields] AND "adult"[Mesh Terms] OR "adult*"[All Fields] | 374 |
| 16 | "infant, very low birth weight"[Mesh Terms] OR "very low birth weight*"[All Fields] OR "very low birthweight*"[All Fields] OR "premature birth"[Mesh Terms] OR "premature birth*"[All Fields] OR "preterm*"[All Fields] AND "exercise"[Mesh Terms] OR "exercise*"[All Fields] OR "physical activ*"[All Fields] AND "adult"[Mesh Terms] OR "adult*"[All Fields] AND humans[Filter] AND english[Filter] | 341 |
| Embase | | |
| # | Searches | Results |
| 1 | exp very low birth weight/ | 16587 |
| 2 | "very low birth weight*".ab,kw,ti. | 10609 |
| 3 | "very low birthweight*".ab,kw,ti. | 2573 |
| 4 | exp prematurity/ | 115544 |
| 5 | "premature birth*".ab,kw,ti. | 7279 |
| 6 | "preterm*".ab,kw,ti. | 121642 |
| 7 | 1 or 2 or 3 or 4 or 5 or 6 | 186421 |
| 8 | exp exercise/ | 389814 |
| 9 | exercise.ab,kw,ti. | 397290 |
| 10 | exp physical activity/ | 486164 |
| 11 | "physical activ*".ab,kw,ti. | 186047 |
| 12 | 8 or 9 or 10 or 11 | 952737 |
| 13 | exp adult/ | 9717583 |
| 14 | "adult*".ab,kw,ti. | 1891556 |
| 15 | 13 or 14 | 10388447 |
| 16 | 7 and 12 and 15 | 806 |
| 17 | limit 16 to (human and english language) | 741 |

Search last updated May 4^th^ 2022. / = Emthree term; ti,ab,kw = title, abstract and keywords fields.
