# Supplementary material for: Physical activity and cognitive function in adults born very preterm or with very low birth weight–an individual participant data meta-analysis
Source: PLoS One. 2024 Feb 13;19(2):e0298311. doi: 10.1371/journal.pone.0298311 (PMC10863878; doi:10.1371/journal.pone.0298311)
Supplement: S2 Table — AYLS = Arvo Ylppö Longitudinal Study; ESTER = ESTER Preterm Birth Study; HeSVA = Helsinki Study of Very Low Birth Weight Adults; NTNU LBW Life = Norwegian University of Science and Technology Low Birth Weight in a Lifetime Perspective study; NZ VLBW = New Zealand Very Low Birth Weight Follow-up Study; Q = question. (DOCX) [file pone.0298311.s003.docx]

**S2 Table. Overview of questions used to assess moderate to vigorous physical activity.**

| Cohort(s) | Question(s) to assess physical activity | Response options | Calculation of self-reported moderate to vigorous physical activity in hours per week |
| --- | --- | --- | --- |
| AYLS and ESTER | Q1. *For how long all together do you do brisk exercise in your spare time?*  Q2*. How much in addition to previous do you do light exercise in your spare time?* | □ *Not at all*  □ *About half an hour per week*  □ *About an hour per week*  □ *About 2-3 h per week*  □ *About 4-6 h per week*  □ *About 7 h or more per week* | Q1 and Q2 were combined |
| HeSVA and NTNU LBW Life | Q1. *How often do you exercise or play sports in your spare time?* | □ *Not at all*  □ *Less than once a month*  □ *1-2 times a month*  □ *Approximately once a week*  □ *2-3 times a week*  □ *4-5 times a week*  □ *Approximately every day* | Participants reporting light running (jogging) and fast running in Q2 were combined with Q1 (frequency) and Q3 (duration). Min/hours per week was recoded into hours per week |
|  | Q2. *Is the exercise you do in your spare time about as demanding as* | □ *Walking*  □ *Alternating walking and light running*  □ *Light running (jogging)*  □ *Fast running* |  |
|  | Q3. *On average, how long does one exercise session, performed in your spare time, take?* | □ *Less than 30 minutes*  □ *30 minutes – less than an hour*  □ *1 hour – less than 2 hours*  □ *2 hours or more* |  |
| NZ VLBW | Q1. *Hours per week moderate physical activity*  Q2. *Hours per week vigorous physical activity* | No response options | Q1 and Q2 were combined |

AYLS = Arvo Ylppö Longitudinal Study; ESTER = ESTER Preterm Birth Study; HeSVA = Helsinki Study of Very Low Birth Weight Adults; NTNU LBW Life = Norwegian University of Science and Technology Low Birth Weight in a Lifetime Perspective study; NZ VLBW = New Zealand Very Low Birth Weight Follow-up Study; Q = question.
