# Supplementary material for: Physical activity and cognitive function in adults born very preterm or with very low birth weight–an individual participant data meta-analysis
Source: PLoS One. 2024 Feb 13;19(2):e0298311. doi: 10.1371/journal.pone.0298311 (PMC10863878; doi:10.1371/journal.pone.0298311)
Supplement: S3 Table — AYLS = Arvo Ylppö Longitudinal Study; ESTER = ESTER Preterm Birth Study; HeSVA = Helsinki Study of Very Low Birth Weight Adults; NTNU LBW Life = Norwegian University of Science and Technology Low Birth Weight in a Lifetime Perspective study; NZ VLBW = New Zealand Very Low Birth Weight Follow-up Study. (DOCX) [file pone.0298311.s004.docx]

**S3 Table. Newcastle-Ottawa criteria and** **quality assessment of each included cohort study.**

**NEWCASTLE - OTTAWA QUALITY ASSESSMENT SCALE FOR COHORT STUDIES**

Note: A study can be awarded a maximum of one star for each numbered item within the Selection and Outcome categories. A maximum of two stars can be given for Comparability

**Selection**

1) Representativeness of the exposed cohort

A) Truly representative of the average ***VP/VLBW (not a sub-selection such as just those with BPD or only males)*** in the community*

B) Somewhat representative of the average ***VP/VLBW*** in the community*

C) Selected group of users e.g. nurses, volunteers

D) No description of the derivation of the cohort

2) Selection of the non-exposed cohort

A) Drawn from the same community as the exposed cohort*

B) Drawn from a different source

C) No description of the derivation of the non-exposed cohort

3) Ascertainment of exposure

A) Secure record (e.g. surgical records)*

B) Structured interview*

C) Written self-report

D) No description

4) Demonstration that outcome of interest was not present at start of study ***(adult physical activity was not known when the participants were recruited)***

A) Yes*

B) No

**Comparability**

1) Comparability of cohorts on the basis of the design or analysis

A) Study has information on ***basic covariates (age and sex)****

B) Study has information on ***basic covariates and mediating variables (full scale IQ and BRIEF-A GEC)****

**Outcome**

1) Assessment of outcome

A) Independent blind assessment*

B) Record linkage*

C) Self-report

D) No description

2) Was follow-up long enough for outcomes to occur ***(Did the cohort assess adult physical activity?)***

A) Yes ***(18 years or older)****

B) No

3) Adequacy of follow up of cohorts

A) Complete follow up - all subjects accounted for*

B) Subjects lost to follow up unlikely to introduce bias - small number lost - >***50%*** (select an

adequate %) follow up, or description provided of those lost)*

C) Follow up rate < ***50%*** (select an adequate %) and no description of those lost

D) No statement

**Quality assessment of each included cohort study.**

| Cohort | Representative-ness of the exposed cohort | Selection of the non-exposed cohort | Ascertainment of exposure | Demonstration that outcome of interest was not present at start of study | Comparability of cohorts on the basis of the design or analysis | Assessment of outcome | Was follow-up long enough for outcomes to occur | Adequacy of follow up of cohorts (above or below 50%) | Overall quality score |
| --- | --- | --- | --- | --- | --- | --- | --- | --- | --- |
| AYLS^[1]^ | A* | A* | A* | A* | A** | C | A* | C | 7 |
| ESTER^[2]^ | A* | A* | A* | A* | A* | C | A* | C | 6 |
| HeSVA^[3]^ | A* | A* | A* | A* | A** | C | A* | B* | 8 |
| NZ VLBW^[4]^ | A* | B | A* | A* | A** | C | A* | B* | 7 |
| NTNU LBW Life^[5]^ | A* | B | A* | A* | A** | C | A* | B* | 7 |

AYLS = Arvo Ylppö Longitudinal Study; ESTER = ESTER Preterm Birth Study; HeSVA = Helsinki Study of Very Low Birth Weight Adults; NTNU LBW Life = Norwegian University of Science and Technology Low Birth Weight in a Lifetime Perspective study; NZ VLBW = New Zealand Very Low Birth Weight Follow-up Study.

# **Table references**

1. Heinonen K, Lahti J, Sammallahti S, Wolke D, Lano A, Andersson S, et al. Neurocognitive outcome in young adults born late-preterm. Dev Med Child Neurol. 2018:267-74. doi: 10.1111/dmcn.13616.

2. Tikanmäki M, Kaseva N, Tammelin T, Sipola-Leppanen M, Matinolli HM, Eriksson JG, et al. Leisure Time Physical Activity in Young Adults Born Preterm. J Pediatr. 2017;189:135-42 e2. doi: 10.1016/j.jpeds.2017.06.068.

3. Kajantie E, Strang-Karlsson S, Hovi P, Räikkönen K, Pesonen A-K, Heinonen K, et al. Adults Born at Very Low Birth Weight Exercise Less than Their Peers Born at Term. J Pediatr. 2010:610-6.e1. doi: 10.1016/j.jpeds.2010.04.002.

4. Yang J, Epton MJ, Harris SL, Horwood J, Kingsford RA, Troughton R, et al. Reduced Exercise Capacity in Adults Born at Very Low Birth Weight: A Population-based Cohort Study. Am J Respir Crit Care Med. 2022;205(1):88-98. doi: 10.1164/rccm.202103-0755OC.

5. Balasuriya CND, Evensen KAI, Mosti MP, Brubakk A-M, Jacobsen GW, Indredavik MS, et al. Peak Bone Mass and Bone Microarchitecture in Adults Born With Low Birth Weight Preterm or at Term: A Cohort Study. J Clin Endocrinol Metab. 2017;102(7):2491-500. doi: 10.1210/jc.2016-3827.
