# Supplementary material for: Physical activity and cognitive function in adults born very preterm or with very low birth weight–an individual participant data meta-analysis
Source: PLoS One. 2024 Feb 13;19(2):e0298311. doi: 10.1371/journal.pone.0298311 (PMC10863878; doi:10.1371/journal.pone.0298311)
Supplement: S4 Table — ISCED = International Standard Classification of Education; NSI = neurosensory impairment from childhood defined as having one or more of the following: visual impairment, hearing impairment, cerebral palsy or cognitive disability (determined through an IQ test or other information); SD = standard deviation; VP/VLBW = very preterm (<32 weeks of gestation)/very low birth weight (<1500g); wk = week. aESTER Preterm Birth Study cohort not included due to no data available for non-participants. bNo data available for the control group in the NZ VLBW cohort. cData missing for 77 participants and 30 non-participants in the VP/VLBW group. dData missing for 39 participants and 42 non-participants in the VP/VLBW group. eData missing for 52 participants and 114 non-participants in the VP/VLBW group, and 98 participants and 223 non-participants in the control group. fData missing for 18 participants and 95 non-participants in the VP/VLBW group, and 17 participants and 141 non-participants in the control group. (DOCX) [file pone.0298311.s005.docx]

**S4 Table.** **Background characteristics of participants and non-participants in the very preterm/very low birth weight and the control group.**

|  | VP/VLBW^a^ | | | | Control^a,b^ | | | |
| --- | --- | --- | --- | --- | --- | --- | --- | --- |
|  | Participants  n=520 | | Non-participants  n=207 | | Participants  n=604 | | Non-participants  n=412 | |
| Birthweight (g), mean (SD) | 1150 | (241) | 1187 | (275) | 3621 | (465) | 3603 | (466) |
| Gestational age (wk), mean (SD) | 29.2 | (2.4) | 29.1 | (2.6) | 39.9 | (1.2) | 39.7 | (1.3) |
| Intraventricular hemorrhage grade 1-4^c^, n (%) | 105 | (20.2) | 45 | (21.7) | - | - | - | - |
| Bronchopulmonary dysplasia^d^, n (%) | 99 | (19.0) | 41 | (19.8) | - | - | - | - |
| Female sex, n (%) | 286 | (55.0) | 84 | (40.6) | 347 | (57.5) | 182 | (44.2) |
| NSI^e^, n (%) | 54 | (10.4) | 18 | (8.7) | 6 | (1.0) | 13 | (3.2) |
| Parental education level^f^, n (%) |  |  |  |  |  |  |  |  |
| Low (ISCED 0-2) | 88 | (16.9) | 30 | (26.8) | 45 | (7.7) | 41 | (15.1) |
| Middle (ISCED 3-5) | 242 | (46.5) | 39 | (34.8) | 225 | (38.3) | 111 | (41.0) |
| High (ISCED 6-8) | 172 | (33.1) | 43 | (38.4) | 317 | (54.0) | 119 | (43.9) |

ISCED = International Standard Classification of Education; NSI = neurosensory impairment from childhood defined as having one or more of the following: visual impairment, hearing impairment, cerebral palsy or cognitive disability (determined through an IQ test or other information); SD = standard deviation; VP/VLBW = very preterm (<32 weeks of gestation)/very low birth weight (<1500g); wk = week.

^a^ESTER Preterm Birth Study cohort not included due to no data available for non-participants.

^b^No data available for the control group in the NZ VLBW cohort.

^c^Data missing for 77 participants and 30 non-participants in the VP/VLBW group.

^d^Data missing for 39 participants and 42 non-participants in the VP/VLBW group.

^e^Data missing for 52 participants and 114 non-participants in the VP/VLBW group, and 98 participants and 223 non-participants in the control group.

^f^Data missing for 18 participants and 95 non-participants in the VP/VLBW group, and 17 participants and 141 non-participants in the control group.
