# Supplementary material for: Physical activity and cognitive function in adults born very preterm or with very low birth weight–an individual participant data meta-analysis
Source: PLoS One. 2024 Feb 13;19(2):e0298311. doi: 10.1371/journal.pone.0298311 (PMC10863878; doi:10.1371/journal.pone.0298311)
Supplement: S5 Table — CI = confidence interval; MVPA = moderate to vigorous physical activity; SD = standard deviation; VP/VLBW = very preterm (<32 weeks of gestation)/very low birth weight (<1500g). aVP/VLBW, Control. bBased on bootstrapped regression analysis with group and cohort as fixed factor, and age and sex as covariates. (DOCX) [file pone.0298311.s006.docx]

**S5 Table.** **Moderate to vigorous physical activity among women and men in the** **very preterm/very low birth weight and the control group.**

|  | Sex | n | VP/VLBW | | | Control | | | n^a^ | Adjusted mean difference (95% CI)^b^ | | p-value |
| --- | --- | --- | --- | --- | --- | --- | --- | --- | --- | --- | --- | --- |
|  |  |  | n | Mean | (SD) | n | Mean | (SD) |  |  |  |  |
| MVPA (hours per week) | Women | 919 | 328 | 2.90 | (3.74) | 591 | 5.88 | (3.31) | 328, 585 | -1.34 | (-1.84 to -0.84) | <.001 |
|  | Men | 724 | 267 | 4.56 | (6.12) | 457 | 6.37 | (3.20) | 267, 449 | -0.86 | (-1.57 to -0.14) | 0.021 |

CI = confidence interval; MVPA = moderate to vigorous physical activity; SD = standard deviation; VP/VLBW = very preterm (<32 weeks of gestation)/very low birth weight (<1500g).

^a^VP/VLBW, Control.

^b^Based on bootstrapped regression analysis with group and cohort as fixed factor, and age and sex as covariates.
