# Supplementary material for: Physical activity and cognitive function in adults born very preterm or with very low birth weight–an individual participant data meta-analysis
Source: PLoS One. 2024 Feb 13;19(2):e0298311. doi: 10.1371/journal.pone.0298311 (PMC10863878; doi:10.1371/journal.pone.0298311)
Supplement: S6 Table — Participants with neurosensory impairment excluded. BRIEF-A GEC = Behavior Rating Inventory of Executive Function–Adult Version, Global Executive Composite (overall summary score); CI = confidence interval; IQ = intelligence quotient; MVPA = moderate to vigorous physical activity; SD = standard deviation; VP/VLBW = very preterm (<32 weeks of gestation)/very low birth weight (<1500g). aVP/VLBW, Control. bBased on bootstrapped regression analysis with group and cohort as fixed factor, and age and sex as covariates. (DOCX) [file pone.0298311.s007.docx]

**S6 Table.** **Moderate to vigorous physical activity, full scale intelligence quotient and Behavior Rating Inventory of Executive Function – Adult Version, Global Executive Composite in the very preterm/very low birth weight and the control group. Participants with neurosensory impairment excluded.**

|  | n | VP/VLBW | | | Control | | | n^a^ | Adjusted mean difference (95% CI)^b^ | | p-value |
| --- | --- | --- | --- | --- | --- | --- | --- | --- | --- | --- | --- |
|  |  | n | Mean | (SD) | n | Mean | (SD) |  |  |  |  |
| MVPA (hours per week) | 1575 | 534 | 3.72 | (5.11) | 1041 | 6.10 | (3.27) | 534, 1028 | -1.05 | (-1.48 to -0.65) | <.001 |
| Full scale IQ | 953 | 382 | 87.4 | (18.0) | 571 | 100.4 | (14.5) | 382, 561 | -12.8 | (-15.4 to -10.2) | <.001 |
| BRIEF-A GEC | 1315 | 438 | 101.4 | (22.2) | 877 | 100.5 | (20.1) | 438, 876 | -0.7 | (-3.5 to 2.2) | .632 |

BRIEF-A GEC = Behavior Rating Inventory of Executive Function – Adult Version, Global Executive Composite (overall summary score); CI = confidence interval; IQ = intelligence quotient; MVPA = moderate to vigorous physical activity; SD = standard deviation; VP/VLBW = very preterm (<32 weeks of gestation)/very low birth weight (<1500g).

^a^VP/VLBW, Control.

^b^Based on bootstrapped regression analysis with group and cohort as fixed factor, and age and sex as covariates.
