# Supplementary material for: Physical activity and cognitive function in adults born very preterm or with very low birth weight–an individual participant data meta-analysis
Source: PLoS One. 2024 Feb 13;19(2):e0298311. doi: 10.1371/journal.pone.0298311 (PMC10863878; doi:10.1371/journal.pone.0298311)
Supplement: S7 Table — Participants with neurosensory impairment excluded. Analyses adjusted for cohort, age and sex. BRIEF-A GEC = Behavior Rating Inventory of Executive Function–Adult Version, Global Executive Composite (overall summary score); CI = confidence interval; IQ = intelligence quotient; VP/VLBW = very preterm (<32 weeks of gestation)/very low birth weight (<1500g). aVP/VLBW, Control. (DOCX) [file pone.0298311.s008.docx]

**S7 Table.** **Direct, indirect and total effect of very preterm/very low birth weight on moderate to vigorous physical activity with cognitive function as mediator. Participants with neurosensory impairment excluded.**

| Model | Cognitive function | n^b^ | Direct effect of VP/VLBW | | Indirect effect of VLBW | | Total effect of VLBW | |
| --- | --- | --- | --- | --- | --- | --- | --- | --- |
|  |  |  | Estimate | 95% CI | Estimate | 95% CI | Estimate | 95% CI |
| 1 | Full scale IQ | 382, 561 | -0.65 | (-1.33 to 0.03) | -0.05 | (-0.29 to 0.18) | -0.70 | (-1.34 to -0.06) |
| 2 | BRIEF-A GEC | 438, 876 | -0.69 | (-1.19 to -0.18) | 0.01 | (-0.04 to 0.06) | -0.67 | (-1.18 to -0.17) |
| 3 | Full scale IQ and BRIEF-A GEC | 362, 522 | -0.61 | (-1.33 to 0.10) | 0.02 | (-0.24 to 0.27) | -0.59 | (-1.27 to 0.08) |

Analyses adjusted for cohort, age and sex.

BRIEF-A GEC = Behavior Rating Inventory of Executive Function – Adult Version, Global Executive Composite (overall summary score); CI = confidence interval; IQ = intelligence quotient; VP/VLBW = very preterm (<32 weeks of gestation)/very low birth weight (<1500g).

^a^VP/VLBW, Control.
