# Supplementary material for: Physical activity and cognitive function in adults born very preterm or with very low birth weight–an individual participant data meta-analysis
Source: PLoS One. 2024 Feb 13;19(2):e0298311. doi: 10.1371/journal.pone.0298311 (PMC10863878; doi:10.1371/journal.pone.0298311)
Supplement: S8 Table — BRIEF-A GEC = Behavior Rating Inventory of Executive Function–Adult Version, Global Executive Composite (overall summary score); CI = confidence interval; EP/ELBW = extremely preterm (<28 weeks of gestation)/extremely low birth weight (<1000g); IQ = intelligence quotient; MVPA = moderate to vigorous physical activity; SD = standard deviation. aEP/ELBW, Control. bBased on bootstrapped regression analysis with group and cohort as fixed factor, and age and sex as covariates. (DOCX) [file pone.0298311.s009.docx]

**S8 Table.** **Moderate to vigorous physical activity, full scale intelligence quotient and Behavior Rating Inventory of Executive Function – Adult Version, Global Executive Composite in the extremely preterm/extremely low birth weight and the control group.**

|  | n | EP/ELBW | | | Control | | | n^a^ | Adjusted mean difference (95% CI)^b^ | | p-value |
| --- | --- | --- | --- | --- | --- | --- | --- | --- | --- | --- | --- |
|  |  | n | Mean | (SD) | n | Mean | (SD) |  |  |  |  |
| MVPA (hours per week) | 1246 | 197 | 2.84 | (4.90) | 1049 | 6.09 | (3.27) | 197, 1034 | -1.60 | (-2.25 to -0.89) | <.001 |
| Full scale IQ | 712 | 136 | 81.8 | (18.8) | 576 | 100.2 | (14.7) | 136, 564 | -18.7 | (-22.4 to -14.8) | <.001 |
| BRIEF-A GEC | 1033 | 151 | 101.1 | (22.2) | 882 | 100.5 | (20.1) | 151, 881 | -1.5 | (-5.5 to 2.6) | .453 |

BRIEF-A GEC = Behavior Rating Inventory of Executive Function – Adult Version, Global Executive Composite (overall summary score); CI = confidence interval; EP/ELBW = extremely preterm (<28 weeks of gestation)/extremely low birth weight (<1000g); IQ = intelligence quotient; MVPA = moderate to vigorous physical activity; SD = standard deviation.

^a^EP/ELBW, Control.

^b^Based on bootstrapped regression analysis with group and cohort as fixed factor, and age and sex as covariates.
