# Supplementary material for: Physical activity and cognitive function in adults born very preterm or with very low birth weight–an individual participant data meta-analysis
Source: PLoS One. 2024 Feb 13;19(2):e0298311. doi: 10.1371/journal.pone.0298311 (PMC10863878; doi:10.1371/journal.pone.0298311)
Supplement: S9 Table — Analyses adjusted for cohort, age and sex. BRIEF-A GEC = Behavior Rating Inventory of Executive Function–Adult Version, Global Executive Composite (overall summary score); CI = confidence interval; EP/ELBW = extremely preterm (<28 weeks of gestation)/extremely low birth weight (<1000g); IQ = intelligence quotient; SD = standard deviation. aEP/ELBW, Control. (DOCX) [file pone.0298311.s010.docx]

**S9 Table.** **Direct, indirect and total effect of extremely preterm/extremely low birth weight on moderate to vigorous physical activity with cognitive function as mediator.**

| Model | Cognitive function | n^a^ | Direct effect of EP/ELBW | | Indirect effect of EP/ELBW | | Total effect of EP/ELBW | |
| --- | --- | --- | --- | --- | --- | --- | --- | --- |
|  |  |  | Estimate | 95% CI | Estimate | 95% CI | Estimate | 95% CI |
| 1 | Full scale IQ | 136, 564 | -0.33 | (-0.74 to 0.08) | -0.07 | (-0.25 to 0.10) | -0.40 | (-0.78 to -0.02) |
| 2 | BRIEF-A GEC | 151, 881 | -0.54 | (-0.86 to -0.23) | 0.01 | (-0.02 to 0.05) | -0.53 | (-0.85 to -0.22) |
| 3 | Full scale IQ and BRIEF-A GEC | 126, 525 | -0.39 | (-0.83 to 0.04) | 0.01 | (-0.18 to 0.20) | -0.38 | (-0.78 to 0.02) |

Analyses adjusted for cohort, age and sex.

BRIEF-A GEC = Behavior Rating Inventory of Executive Function – Adult Version, Global Executive Composite (overall summary score); CI = confidence interval; EP/ELBW = extremely preterm (<28 weeks of gestation)/extremely low birth weight (<1000g); IQ = intelligence quotient; SD = standard deviation.

^a^EP/ELBW, Control.
